# Research on the effect of post-activation potentiation under different velocity loss thresholds on boxer’s punching ability

**DOI:** 10.3389/fphys.2024.1429550

**Published:** 2024-10-11

**Authors:** Weijia Cui, Yiming Chen, Dexin Wang

**Affiliations:** ^1^ School of Athletic Performance, Shanghai University of Sport, Shanghai, China; ^2^ School of Exercise and Health, Shanghai University of Sport, Shanghai, China

**Keywords:** boxing, punching ability, post-activation potentiation, velocity-based training, velocity loss

## Abstract

This study was conducted in accordance with the principles of velocity-based training theory, with the objective of investigating the effects of post-activation potentiation (PAP) induced by different velocity loss (VL) thresholds (10% vs. 20%) on the punching ability of boxers. In addition, the aim was to determine the velocity loss thresholds and time nodes that produced the optimal activation effect. Twenty-four male elite boxers were randomly assigned to three groups: CON, 10 VL, and 20 VL. All subjects in the three groups underwent an activation intervention involving an 85% of the one-repetition maximum (1RM) squat, with 6-8 repetitions performed in the CON. The number of repetitions in the 20%VL and 10 VL was determined based on the velocity loss monitored by the GymAware PowerTool system. Four time points were selected for observation: the 4th, 8th, 12th and 16th minutes. These were chosen to test the subjects’ punching ability. The results demonstrated that activation training at different VL induced a post-activation potentiation in boxers, improving punching ability bilaterally and to a greater extent than in the CON. The dominant side demonstrated the greatest efficacy at the 12th minute under the 20% velocity loss threshold, while the non-dominant side exhibited the greatest efficacy at the 8th minute under the 10% velocity loss threshold.

## 1 Introduction

Boxers must possess excellent punching ability in order to deliver high-quality and effective punches (Loturco et al., 2016). A warm-up based on post-activation potentiation (PAP) may have a superior optimisation effect on boxers’ punching ability, thus further improving performance. In the existing studies, the activation training protocols have used the percentage of the one-repetition maximum (1RM) as the loading intensity and a fixed number of repetitions as the loading volume (Guo et al., 2022). However, such programmes ignore individual differences in athletes and fluctuations in physical status. Due to pre-competition physiological and psychological changes, as well as sleep status, fatigue recovery, nutritional supplementation and other factors, the 1RM of athletes is not stable and it is difficult to determine the optimal number of repetitions (González-Badillo and Sánchez-Medina, 2010). This makes it impossible to precisely control the load intensity and load volume during the induction of the PAP, which is not conducive to the production of an optimal PAP, or even fatigue, which is counterproductive (Dorrell et al., 2020; Liao et al., 2021). Therefore, further investigation is required into the loading arrangement for inducing the PAP.

Some researchers have attempted to investigate the regulation of strength training loads using movement velocity as a point of departure. Their findings indicate that the load intensity at the time of completing the movement was significantly correlated with the movement velocity in a variety of types of strength training (Pereira and Gomes, 2003; Weakley et al., 2021), and significantly correlated with the percentage of velocity loss (VL) (González-Badillo et al., 2006; Pareja-Blanco et al., 2017b). The amount of load in strength training should not only be a fixed number of repetitions corresponding to the relative load intensity. In order to ensure the relevance and effectiveness of the load arrangement, it is recommended that the load be regulated according to the size of the VL in each set of training. Not only can the target training intensity be achieved, but also the level of fatigue can be monitored in real time to prevent overfatigue, thus achieving the best training effect (Sánchez-Medina and González-Badillo, 2011; Schilling et al., 2008).

Consequently, the percentage of VL can be employed as a load modifying variable in strength training for the purpose of inducing PAP. Existing studies have demonstrated that 20% VL represents a critical value below which explosive power is favoured, and above which it is more favourable for musCIe hypertrophy (Pareja-Blanco et al., 2020). Nevertheless, studies have also indicated that 10% VL is more favourable for explosive power gains than 20% VL. A study found that while both 10% and 20% VL programs led to similar strength gains, the 10% VL condition provided better outcomes in explosive power due to reduced fatigue levels (Krzysztofik et al., 2022). Research has shown that lower VL thresholds, such as 10% VL, may be more effective for maximizing explosive power gains compared to higher thresholds like 20% VL. This is because less fatigue is accumulated with lower VL, allowing for better power output during subsequent efforts (Andersen et al., 2024).

However, there is still a lack of extensive research applying these methods specifically to boxing programs, and more studies are needed to establish the superiority (or lack thereof) of VL-induced PAP compared to traditional PAP methods (González-Badillo et al., 2022). Therefore, the present study investigated the effects of PAP on punching ability in boxers based on different VLs.

### 1.1 Aim and objectives

In this study, three different PAP training protocols with different loads were designed and tested on the boxers’ punching ability sub-indicators at different recovery times to compare and analyse the effects of three different PAP training protocols, namely, 10%VL and 20%VL, and the traditional protocol (based on repetitions of 1%1RM), on punch force, speed and power, so as to investigate the optimal VL threshold and recovery time.

The present study expects to optimise athletic performance and promote punching ability in boxers by exploring an intervention protocol focusing on load scheduling refinement and individualisation. It also promotes the further application of the activation modality of the PAP in practice, provides data support and theoretical basis for the training load arrangement of the boxers’ pre-fight warm-up programme and the PAP in their daily training, and provides data support and theoretical basis for in-depth research in this field.

## 2 Materials and methods

### 2.1 Sample and participants

Twenty-four male elite boxers (age: 19.14 ± 1.82; height: 174.52 ± 3.76 cm; body weight: 64.75 ± 6.57 kg; 1RM squat: 118.62 ± 11.66 kg; sport level: national) consented to participate in this study. Participants reported no history of knee injury within 6 months prior to the testing. They were informed about the procedure and the aim of the study, and subsequently they provided their written consent for participation. Ethical consent was provided by Shanghai University of Sport research ethics committee (approval number: 102772023RT153) and in accordance with the Helsinki deCIaration.

### 2.2 The main experimental steps

#### 2.2.1 Experimental equipment

In this study, the athlete’s punching ability was measured by StrikeTec (Striketec Sensor Kit, StrikeTec, TX, United States). It integrates inertial measurement unit (IMU) technology, using accelerometers and gyroscopes to capture real-time data on punch force, speed and power. The sensors are installed in boxing gloves, and the collected data is transmitted via Bluetooth to a mobile app for analysis and visualization (Menzel and Potthast, 2021a; Menzel and Potthast, 2021b).

Velocity loss was derived by GYM (GymAware Power Tool, Kinetic Performance Technologies, Australia), recording the velocity of barbell movement during the squat. It operates by utilizing a linear position transducer to measure bar velocity during resistance training exercises. The device is attached to the barbell or weight, and as the athlete performs lifts, it captures real-time data on movement velocity production. This data is transmitted to a mobile app, where it is analyzed and visualized (Dorrell et al., 2019; Orange et al., 2020).

#### 2.2.2 Experimental methods

##### 2.2.2.1 Velocity loss test

The lower limb muscle fatigue was monitored in real time by monitoring the velocity of barbell movement during exercise with the GYM. In this study, the 10VL, 20VL, and CON all used 85% 1RM as the loading intensity (Garbisu-Hualde and Santos-Concejero, 2021), and in the selection of loading volume, the 10VL and 20VL monitored the loading through the real-time feedback from the GYM, and determined the loading volume (the number of repetitions) based on the velocity loss in the feedback data, and the CON used 6-8 repetitions as the loading volume.

The GYM was prepared and placed on the side of the squat rack prior to the start of the test, and the subject’s body weight and 85% of the 1RM weight were entered into the data terminal. The sensing cable of the GYM was then connected to the barbell end and manually zeroed in the data terminal. The test required the subject to perform three squats at 85% of the 1RM load intensity, squatting until the knee was bent at slightly more than 90°, or until the thighs were parallel to the floor, pausing for one second, and then squatting quickly to the starting position. The maximum value of the average squat velocity over the three sessions was recorded as the average squat velocity during formal training using real-time feedback data from the GYM. During the formal training intervention, if the subject’s velocity per squat was 90%–100% (10VL) and 80%–100% (20VL) of the measured mean velocity, training continued, and when the mean velocity per squat was lower than 90% (10VL) and 80% (20 VL) of the measured mean velocity, i.e., the velocity loss was more than 10% (10VL) and 20% (20VL), the training was ended.

##### 2.2.2.2 Punching ability test

Subjects were required to wear uniform boxing gloves. Before the start of the test, the chip in the StrikeTec device was placed on the outside of the subject’s wrist joint and secured with a strap. The data terminal was zeroed, the subject’s height, weight, and age were entered, and the subject was explained the requirements of the test maneuvers. The punch requires the subject to freely control the distance between the sandbag and the subject, and under the condition of full stomping and force generation, the subject can take a step up to punch the sandbag with full force. The interval between single punch was 15 s. After completing 5 punches and generating valid data, the maximum and minimum values in the data were excluded, and the average value of the remaining 3 punches was taken as the final result. The average punch power data comes from the product of average punch speed and average punch force. During the testing process, the staff should monitor the quality of the subject’s punches to prevent ineffective punches. If there is an error in the action need to be re-punch after an interval of 15 s, until the end of the test.

#### 2.2.3 Eexperimental procedure

**FIGURE 1:**
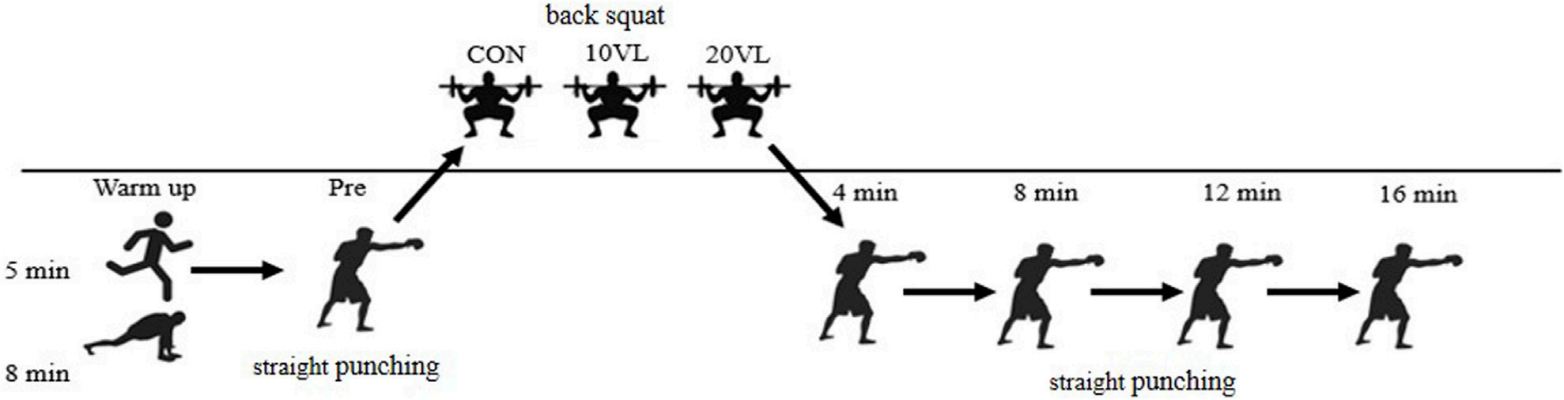
Flowchart of the experiment.

The experimental procedure is shown in Figure 1. The subjects were randomly assigned to three groups: CON, 10VL, and 20VL. The 85% 1RM squat was selected as the activation movement. At the beginning of the experiment, subjects were asked to perform a 5-min jogging warm-up as well as dynamic stretching, followed by an 8-min boxing-specific warm-up, after which the pre-test began.

##### 2.2.3.1 CON

The CON performed a punching ability pre-test after completing the warm-up, with a 5 min interval before completing a pre-warm-up of 85% 1RM x 3 reps of squats, and then began the formal workout 4 min later by completing 85% 1RM x (6–8) reps of squats. The formal workout entailed completing two sets with a 2-min interval between sets. Striking ability tests were performed at four time points, 4, 8, 12, and 16 min after completion of the formal training, and the results of the subjects’ punch force, punch speed, and punch power data were recorded.

##### 2.2.3.2 10VL

The 10VL underwent a pre-test of punching ability after completing the warm-up, and after a 5-min interval, completed a pre-warm-up of 85% of 1RM x 3 repetitions of squats, while the highest average velocity during the three squats was recorded using the GYM. Formal training began 4 min later, and subjects were required to complete 85% of 1RM squats, with the number of repetitions, i.e., the amount of load, based on the number of repetitions, i.e., the load, was based on the loss of velocity as measured by the GYM, and the training was completed when the loss of velocity exceeded the 10% threshold. Formal training required the completion of two sets with a 2-min interval between sets. The punching ability test was conducted at the 4th, 8th, 12th, and 16th minutes after the completion of the formal training, and the results of the subjects’ punch force, punch speed, and punch power data were recorded.

##### 2.2.3.3 20VL

The 20VL underwent a pre-test of punching ability after completing the warm-up, and after a 5-min interval, completed a pre-warm-up of 85% of 1RM x 3 repetitions of squats, while the highest average velocity during the three squats was recorded using the GYM. Formal training began 4 min later, and subjects were required to complete 85% of 1RM squats, with the number of repetitions, i.e., the amount of load, based on the number of repetitions, i.e., the load, was based on the loss of velocity as measured by the GYM, and the training session ended when the loss of velocity exceeded the 20% threshold. Formal training required the completion of two sets with a 2-min interval between sets. The punching ability test was conducted at the 4th, 8th, 12th, and 16th minutes after the completion of the formal training, and the results of the subjects’ punch force, punch speed, and punch power data were recorded.

#### 2.2.4 Outcome measures

Squat maximum strength: 1RM squat (SQ), Velocity loss (VL, velocity is the rate of change of an object’s position, including direction and magnitude): barbell movement velocity during squat. Punching ability: punch force (PF, force is an interaction that changes the motion of an object when unopposed), punch speed (PS, speed is how fast an object is moving, measured as distance traveled per unit time), and punch power (PP, power is the rate at which work is done or energy is transferred) on dominant and non-dominant sides (Smith et al., 2000).

### 2.3 Statistical analysis

Descriptive statistics (means and standard deviations) were performed to summarize all data. Two-way analysis of variance (ANOVA) were conducted (time*group), with the between-group factor being the effect of grouping (CON, 10VL, 20VL) and the within-group factor being the measurement time (PRE, 4 min, 8 min, 12 min, 16 min). The data in each group exhibited a normal distribution, as indicated by the Shapiro-Wilk (S-W) test. The data were tested for sphericity using repeated measures ANOVA. If *p* > 0.05, spherical symmetry was met. Conversely, if *p* < 0.05, the results of the multivariate test prevailed. When there was an interaction effect between two factors, a simple effects analysis was performed for each factor. The strength of association within groups was evaluated by calculating the partial eta square (*η* ^2^) separate effect size. The larger the *η* ^2^ value, the larger the magnitude of the difference. Finally, the optimal VL and recovery time points for the PAP of boxers’ punching ability were determined following multiple comparisons to analyse the between-group differences under the same load, as well as to compare the various time periods under that load with the immediate aftermath of the exercise (with the significance level taken as *p* < 0.05). All statistical analyses were performed using PRISM (GraphPad Software, Inc. Version prism 8.0 for Windows) and SPSS 26.0 software (SPSS Inc., Chicago, IL, United States).

Utilizing G*Power 3.1 software, we took a moderate effect size (*η* ^2^ = 0.059), with a statistical power of 0.8 and a significance level of 0.05. Derived the need for a minimum of 24 subjects.

## 3 Results

### 3.1 Effect of different velocity loss thresholds on punching speed

Punching speed refers to the velocity at which a punch is delivered, typically measured in meters per second (m/s). It reflects the ability of the boxer to execute punches rapidly, which is crucial for both offensive and defensive maneuvers in boxing (Horan and Kavanagh, 2012).

**FIGURE 2:**
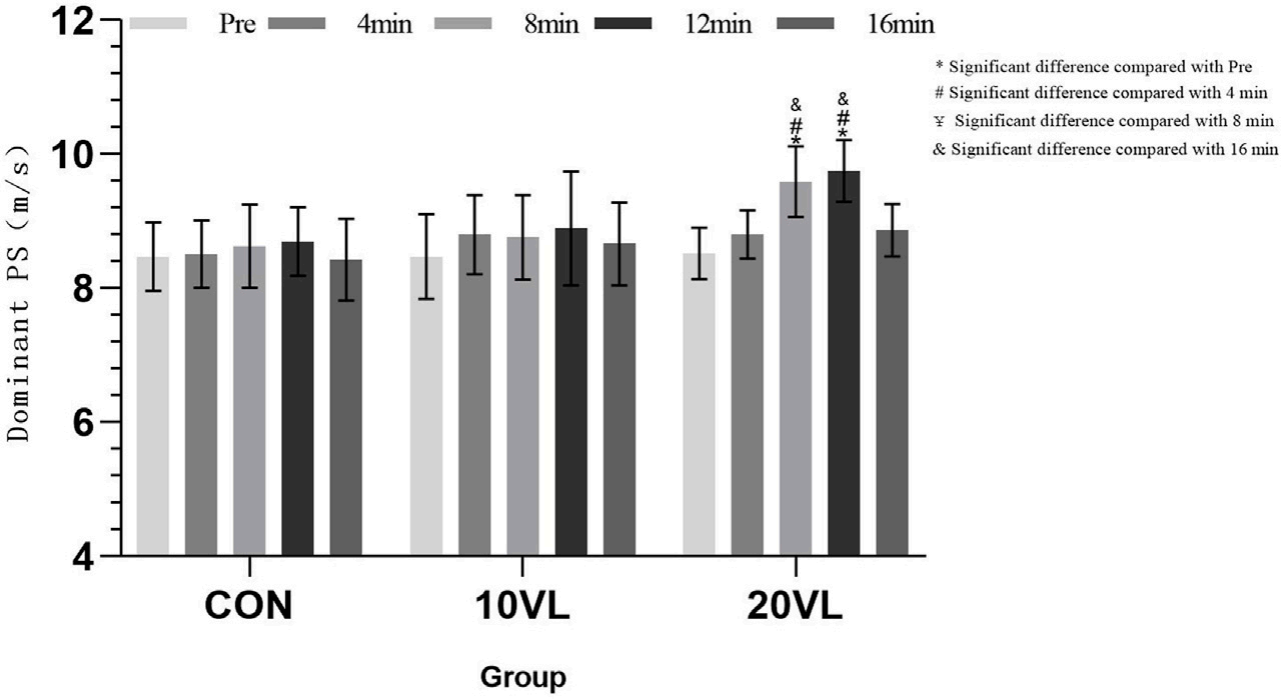
Variation in punching speed of the dominant side at different time points.

Table 1 shows the results of punching speed, Figure 2 shows the variation of punching speed on the dominant side at different time points.

**TABLE 1:** Results of punching speed (M±SD, m/s).

	Group	PRE	4 min	8 min	12 min	16 min
Dominant	CON	8.47 ± 0.51	8.51 ± 0.5	8.63 ± 0.62	8.7 ± 0.51	8.43 ± 0.61
	10VL	8.47 ± 0.63	8.8 ± 0.59	8.76 ± 0.63	8.89 ± 0.85	8.66 ± 0.62
	20VL	8.52 ± 0.38	8.8 ± 0.36	9.59 ± 0.53	9.75 ± 0.46	8.87 ± 0.39
Non-dominant	CON	6.72 ± 0.19	6.43 ± 1.00	6.77 ± 0.29	6.64 ± 0.52	6.57 ± 0.51
	10VL	6.76 ± 0.23	6.74 ± 0.94	7.35 ± 0.52	7.28 ± 0.33	6.92 ± 0.39
	20VL	6.76 ± 0.22	6.56 ± 0.94	6.68 ± 0.59	6.76 ± 0.63	6.94 ± 0.88

ANOVA results showed that a significant group*time interaction could be observed on the dominant side (*F* = 3.659, *p* < 0.05, *η* ^2^ = 0.125), and a significant main effect of time could be observed (*F* = 11.603, *p* < 0.001, *η* ^2^ = 0.185). 20VL: Compared to Pre, 8 min (*p* < 0.05, 95% CI: −1.629 to −0.525) and 12 min (*p* < 0.05, 95% CI: −1.793 to −0.668) increased; 8 min (*p* < 0.05, 95% CI: −13.67 to −0.21) and 12 min (*p* < 0.05, 95% CI: −1.494 to −0.391) increased compared to 4 min; 16 min decreased compared to 8 min (*p* < 0.05, 95%CI: −1.305 to −0.146) and 12 min (*p* < 0.05, 95% CI: −1.361 to −0.397).

**FIGURE 3:**
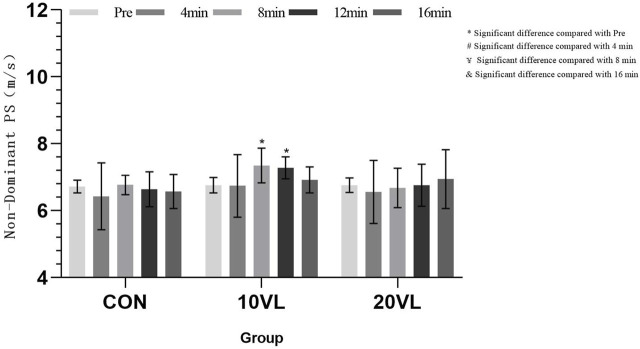
Variation in punching speed of the non-dominant side at different time points.

Figure 3 shows the variation of punching speed on the non-dominant side at different time points.

A significant group*time interaction could be observed on the non-dominant side (*F* = 2.885, *p* < 0.05, *η* ^2^ = 0.191), and a significant main effect of time could be observed (*F* = 3.666, *p* < 0.05, *η* ^2^ = 0.234). 10VL: Compared to Pre, 8 min (*p* < 0.05, 95% CI: −0.991 to −0.2) and 12 min (*p* < 0.05, 95% CI: −0.923 to −0.123) increased.

### 3.2 Effect of different velocity loss thresholds on punching force

Punching force is the amount of force exerted upon impact with a target, often measured in Newtons (N). It is influenced by the mass of the fist and the acceleration of the punch, adhering to Newton’s second law of motion (Force = Mass × Acceleration) (Olberding and Deban, 2017).

**FIGURE 4:**
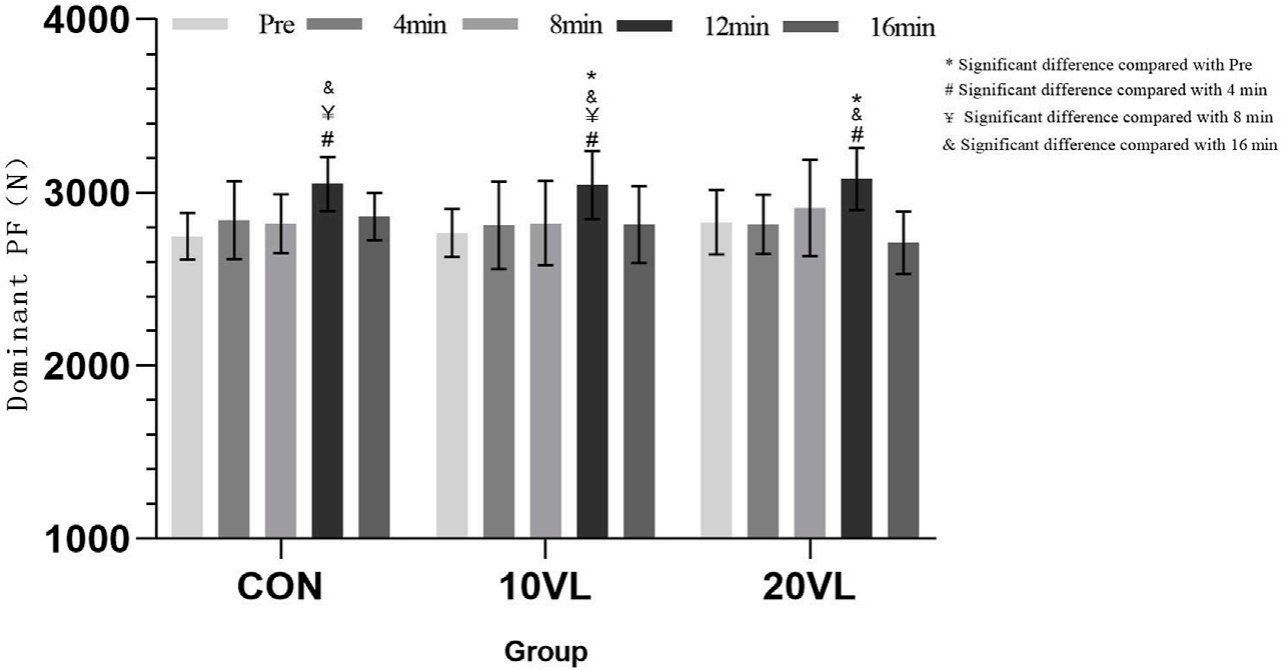
Variation in punching force of the dominant side at different time points.

Table 2 shows the results of punching force, Figure 4 shows the variation of the dominant side’s punching force at different time points.

**TABLE 2:** Results of punching force (M±SD, N).

	Group	PRE	4 min	8 min	12 min	16 min
Dominant	CON	2,747.71 ± 134.89	2,840.32 ± 224.41	2,820.67 ± 169.72	3,048.41 ± 156.23	2,860.15 ± 135.84
	10VL	2,765.33 ± 138.77	2,809.11 ± 252.2	2,822.67 ± 243.69	3,043.3 ± 197.11	2,815.28 ± 222.49
	20VL	2,827.36 ± 186.15	2,816.49 ± 171.34	2,910.67 ± 277.51	3,079.39 ± 178.5	2,709.46 ± 179.8
Non-dominant	CON	2,270.86 ± 113.43	2,361.86 ± 220.77	2,456.06 ± 177.01	2,468.14 ± 220.79	2,395.16 ± 197.94
	10VL	2,263.42 ± 113.37	2,429.71 ± 200.35	2,552.71 ± 159.23	2,418.91 ± 187.42	2,249.95 ± 161.7
	20VL	2,318.88 ± 80.85	2,367.5 ± 232.75	2,541.57 ± 186.33	2,426.73 ± 180.32	2,267.18 ± 181.06

No significant group*time interaction was observed on the dominant side (*F* = 1.352, *p* > 0.05, *η* ^2^ = 0.05), but a significant main effect of time could be observed (*F* = 19.454, *p* < 0.001, *η* ^2^ = 0.276). CON: 12 min compared to 4 min (*p* < 0.05, 95% CI: −398.92 to −17.258) and 8 min (*p* < 0.05, 95% CI: −397.88 to −57.591) increased; 16 min decreased compared to 12 min (*p* < 0.05, 95% CI: −348.13 to −28.375). 10VL: 12 min increased compared to Pre (*p* < 0.05, 95% CI: −449.74 to - 106.2), 4 min (*p* < 0.05, 95% CI: −425.02 to −43.358) and 8 min (*p* < 0.05, 95% CI: −390.77 to −50.48); 16 min decreased compared to 12 min (*p* < 0.05, 95% CI: −387.9 to −68.138). 20VL: 12 min increased compared to Pre (*p* < 0.05, 95% CI: −423.8 to −80.26) and 4 min (*p* < 0.05, 95% CI: −453.73 to −72.073); 16 min decreased compared to 12 min (*p* < 0.05, 95% CI: −529.81 to −210.05).

**FIGURE 5:**
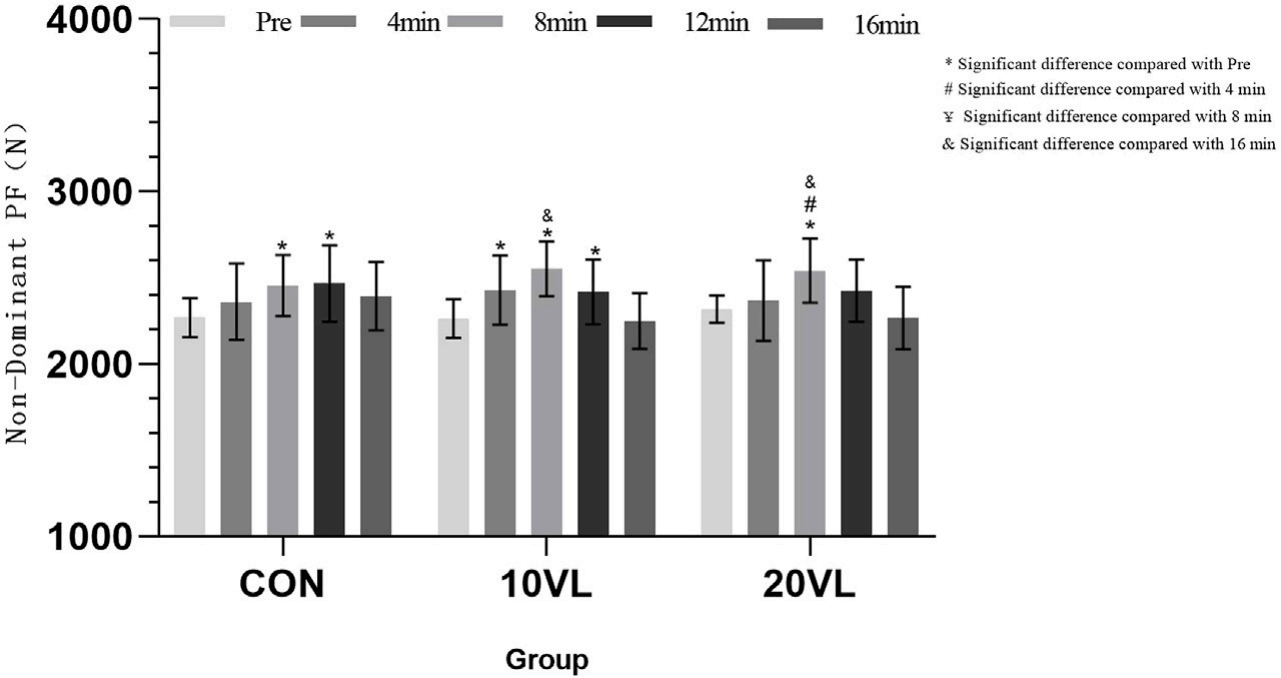
Variation in punching force of the non-dominant side at different time points.

Figure 5 shows the change in punching force on the non-dominant side at different time points.

No significant group*time interaction was observed on the non-dominant side (*F* = 1.636, *p* > 0.05, *η* ^2^ = 0.118), but a significant main effect of time was able to be observed (*F* = 30.883, *p* < 0.001, *η* ^2^ = 0.72). CON: Compared to Pre, 8 min (*p* < 0.05, 95% CI: −315.27 to −55.129) and 12 min (*p* < 0.05, 95% CI: −346.94 to −47.614) increased. 10VL: Compared to Pre, 4 min (*p* < 0.05, 95% CI: −332.64 to 0.071), 8 min (*p* < 0.05, 95% CI: −419.36 to −159.22) and 12 min (*p* < 0.05, 95% CI: −305.15 to −5.823) increased; 16 min decreased compared to 8 min (*p* < 0.05, 95% CI: −467.89 to −137.64). 20VL: 8 min increased compared to Pre (*p* < 0.05, 95% CI: −352.75 to −92.617) and 4 min (*p* < 0.05, 95% CI: −329.79 to −18.347); 16 min decreased compared to 8 min (*p* < 0.05, 95% CI: −439.52 to −109.26).

### 3.3 Effect of different velocity loss thresholds on punching power

Punching power combines both speed and force to quantify the effectiveness of a punch. It is often described as the ability to deliver a powerful strike capable of generating significant impact energy, typically measured in Watts (W) or calculated using the formula Power = Force × Speed (Cormie et al., 2010).

**FIGURE 6:**
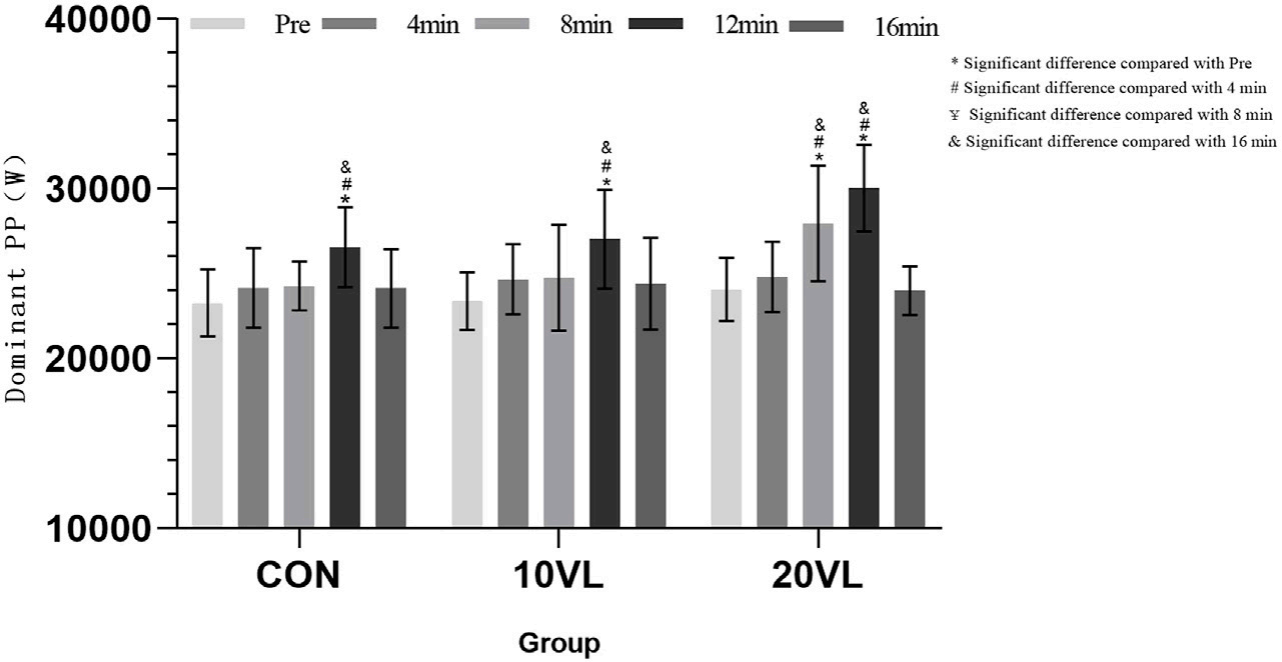
Variation in punching power of the dominant side at different time points.

Table 3 shows the results of punching power, Figure 6 shows the variation of the punching power of the dominant side at different time points.

**TABLE 3:** Results of punching power (M±SD, W).

	Group	Pre	4 min	8 min	12 min	16 min
Dominant	CON	23,274.03 ± 1980.7	24,158.92 ± 2,357.08	24,273.88 ± 1,431.94	26,560.06 ± 2,353.01	24,127.27 ± 2,316.2
	10VL	23,388.04 ± 1707.93	24,665.3 ± 2062.62	24,757.17 ± 3,121.62	27,027.17 ± 2,903.33	24,401.17 ± 2,701.06
	20VL	24,073.61 ± 1857.87	24,818.83 ± 2077.41	27,959.35 ± 3,403.33	30,033.75 ± 2,546.94	23,994.94 ± 1,434.54
Non-dominant	CON	15,253.66 ± 801	15,218.72 ± 2,840.98	16,621.81 ± 1,479.4	16,366.68 ± 1725.37	15,726.5 ± 1717.08
	10VL	15,300.22 ± 987.12	16,335.81 ± 2,396.31	18,751.11 ± 1,469.64	17,641.08 ± 1852.45	15,549.81 ± 1,215.71
	20VL	15,664.97 ± 701.42	15,494.14 ± 2,490.46	16,994 ± 1977.05	16,433.52 ± 2,309.82	15,678.82 ± 2064.1

A significant group*time interaction could be observed on the dominant side (*F* = 3.251, *p* < 0.05, *η* ^2^ = 0.113) and a significant main effect of time could be observed (*F* = 28.448, *p* < 0.001, *η* ^2^ = 0.358). CON: 12 min increased compared to Pre (*p* < 0.05, 95% CI: −5,361.98 to −1,210.09) and 4 min (*p* < 0.05, 95% CI: −4,673.86 to −128.433); 16 min decreased compared to 12 min (*p* < 0.05, 95% CI: −4,573.44 to −292.143). 10VL: 12 min increased compared to Pre (*p* < 0.05, 95% CI: −5,715.08 to −1,563.19) and 4 min (*p* < 0.05, 95% CI: −4,634.59 to −89.162); 16 min decreased compared to 12 min (*p* < 0.05, 95% CI: −4,766.65 to −485.353). 20VL: Compared to Pre, 8 min (*p* < 0.05, 95% CI: −6,397.39 to −1,374.09) and 12 min (*p* < 0.05, 95% CI: −8,036.09 to −3,884.2) increased; compared to 4 min, 8 min (*p* < 0.05, 95% CI: −5,682.62 to −598.418) and 12 min (*p* < 0.05, 95% CI: −7,487.64 to −2,942.21) increased; 16 min decreased compared to 8 min (*p* < 0.05, 95% CI: −6,629.4 to −1,299.43) and 12 min (*p* < 0.05, 95% CI: −8,179.47 to −3,898.17).

**FIGURE 7:**
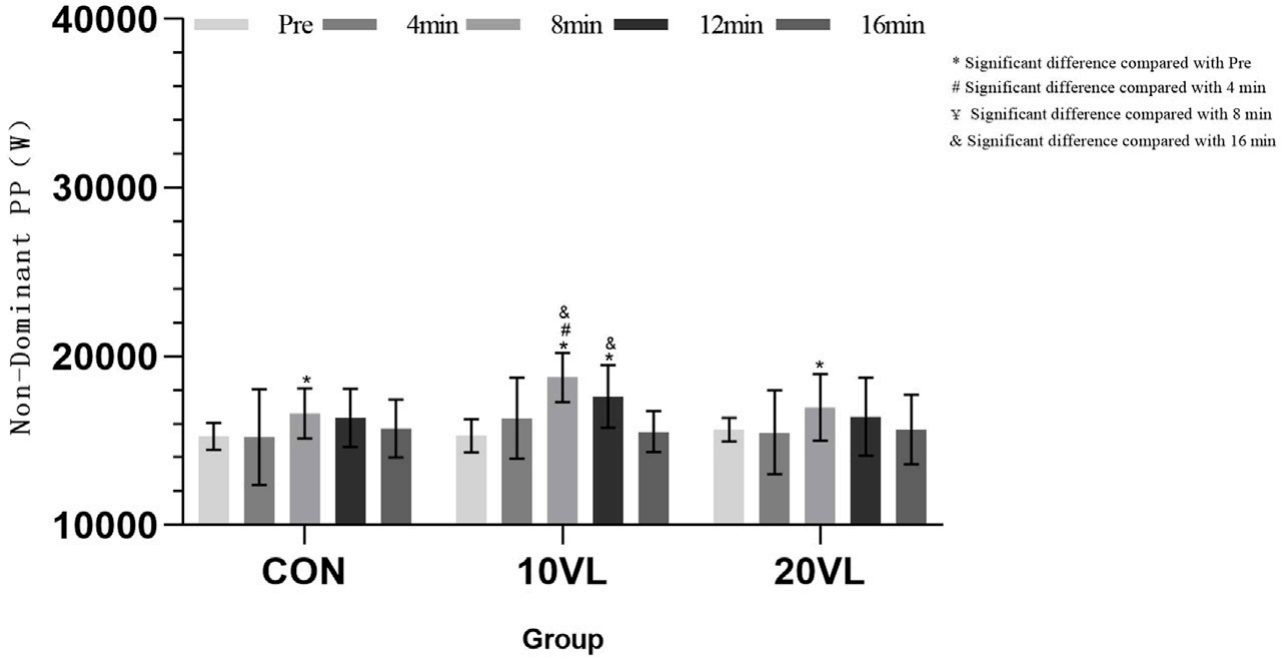
variation of punching power on the non-dominant side at different time points.

Figure 7 shows the variation of punching power on the non-dominant side at different time points.

The non-dominant side was able to observe a significant group*time interaction (*F* = 2.483, *p* < 0.05, *η* ^2^ = 0.169) and was able to observe a significant main effect of time (*F* = 24.782, *p* < 0.001, *η* ^2^ = 0.674). CON: 8 min increased compared to Pre (*p* < 0.05, 95% CI: −2,628.91 to −107.386). 10VL: Compared to Pre, 8 min (*p* < 0.05, 95% CI: −4,711.65 to −2,190.13) and 12 min (*p* < 0.05, 95% CI: −3,828.52 to −853.199) increased; 8 min increased compared to 4 min (*p* < 0.05, 95% CI: −4,380.14 to −450.447); 16 min decreased compared to 8 min (*p* < 0.05, 95% CI: −4,906.01 to −1,496.58) and 12 min (*p* < 0.05, 95% CI: −3,683.3 to −499.243). 20VL: 8 min increased compared to Pre (*p* < 0.05, 95% CI: −2,589.79 to −68.272).

## 4 Discussion

This study represents the inaugural investigation into the impact of PAP induced by varying VL on the punching ability of boxers. The study employed a three-pronged approach, encompassing the examination of the load stimulus, fatigue effect and fatigue recovery time at distinct VL. The objective was to identify the optimal PAP, as well as the VL and time points that would induce this effect.

### 4.1 Post-activation enhancement effect on punching ability at different velocity loss thresholds

Current research in this area suggests that 10VL and 20VL may be better for athletes who need explosive power, but it is not known which is more appropriate for boxers (Pérez-Castilla et al., 2018).

We found that the 10VL and the 20VL exhibited significantly enhanced punching ability compared to the CON. However, the magnitude of change in various sub-indicators of punching ability at different VL varied. Furthermore, the study revealed a significant asymmetry between the punching abilities of the dominant and non-dominant sides. The punching ability of the dominant side was found to be significantly superior to that of the non-dominant side at different VL. This is attributed to the fact that boxing techniques encompass forehands and backhands, and that long-term training tends to result in uneven musCIe strength on both sides of the body. Boxers tend to utilise the dominant side with greater frequency in order to land as many effective punches as possible. Although the data indicates that the punching ability of the dominant side is significantly superior to that of the non-dominant side, which appears to be more conducive to effective punching, it is important to recognise the value of training the non-dominant side. This is due to the complexity of boxing technical movements, the uncertainty of the use of technical and tactical skills, and the transition between attack and defence in the ring.

We therefore discuss the differences between the dominant and non-dominant sides at different VLs separately and explore the reasons for the differences.

The present study demonstrated that the PAP induced by 20VL was beneficial in increasing the punching speed of the dominant side of the subjects. Compared with the CON and the 10VL, the 20VL exhibited the most favourable outcomes, likely due to the load effectively improving the neuromuscular coordination of the subjects’ organisms and facilitating greater fast musCIe fibre recruitment (Pareja-Blanco et al., 2017b; Sañudo et al., 2020). The dynamics of the kinetic chain necessitate that a straight punch requires the lower extremity to generate force from the stirrups, which is then transferred from the core to the arm to complete the end release of the force. Consequently, the amount of force generated by the lower limb stirrups will have a direct impact on the rate of growth of the force, and thus on the speed of the punch at the time of release of the upper limb. The 20% VL can enhance the neural control of musCIes in the lower limb stirrups (Enoka, 2012; Heckman and Enoka, 2012), which is conducive to the improvement of punching speed. Furthermore, the velocity of power transmission within the kinetic chain is also influenced by the excitation and inhibition of the motor nerve centre within the cerebral cortex and the coordination between the upper and lower limbs. Existing studies have confirmed that the PAP induced at the threshold of 20% loss of velocity can optimise the effect of these factors (González-Badillo et al., 2011).

The PAP induced by the 20VL in the present study was able to increase the punching force of the subjects. This is likely due to the fact that the activation training at this load effectively increased the sensitivity of calcium ions in the myocytes, which led to the enhancement of the fast musCIe contraction. At the same time, by changing the pinnation angle, the musCIe contraction force was enhanced (Zimmermann et al., 2020). The recruitment of more fast musCIe fibres was facilitated by 20% VL, as the preliminary activation training increased neural excitability and accelerated the conduction rate of action potentials in the nerves. This enabled the body to recruit more fast motor units (Pareja-Blanco et al., 2017a), which ultimately manifested itself in the enhancement of the subject’s punching force. This hypothesis has been corroborated by previous studies, inCIuding those conducted by Galiano et al. who conCIuded that 20% VL was efficacious in augmenting maximal strength and lower limb explosive power (Galiano et al., 2022), and Folland and Santanielo et al. who demonstrated that 20% VL was beneficial in promoting changes in the pinnation angle of the musCIe fibres, thereby increasing the force of the musCIe contraction (Folland and Williams, 2007; Santanielo et al., 2020).

Given that punch power is positively correlated with punch force and punch speed, the results of the study demonstrated consistency between the variables of PP and PS and PF. The maximum values were observed on the dominant side at 12 min in the 20VL and on the non-dominant side at 8 min in the 10VL.

### 4.2 Post-activation enhancement effects at different velocity loss thresholds at different time points

Most of the studies suggested that the PAP occurs at 4 min post-intervention, and the following 8 min, 12 min and 16 min were used as the observation points (Gilbert and Lees, 2005), but in practice we found that the time point at which the optimal PAP occurs varies between VLs.

The PAP undergoes changes at different time points due to the generation and dissipation of fatigue. Following activation training, the PAP is induced while fatigue ensues due to the stimulation of the body by the load, and there is a dynamic equilibrium between the PAP and the fatigue effect. In this study, the peak is the point in time when the fatigue effect is minimal and the PAP is optimal. In rugby, which also requires lower body explosive power, the researchers observed that the level of lower body explosive power increased significantly at 8 and 12 min post-intervention. This indicated that the PAP was more pronounced at 8–12 min intervals (Kilduff et al., 2007). These findings are consistent with those of the present study, which demonstrated that the bilateral punching ability of the groups exhibited a significant enhancement at both time nodes. The dominant side, 20VL, exhibited the most pronounced PAP at 12 min, while the non-dominant side, 10VL, demonstrated the optimal PAP at 8 min.

The duration of fatigue recovery is directly proportional to the intensity of the load stimulus to which the organism is exposed. Consequently, the interval time between activation training and testing exerts a significant influence on the acute enhancement effect of the PAP on the organism. If the interval time is too short, the organism is in a state of fatigue, and the fatigue effect is greater than the PAP, which is not conducive to the enhancement of athletic performance. Conversely, if the interval time is too long, the fatigue effect is gradually decreasing, but the PAP will also subside. This is why the results of the present study show a rising-deCIining wave pattern at each time point.

In terms of punching speed on the dominant side, the 20VL demonstrated a significant increase at 8 min and reached a peak at 12 min. This suggests that the activation effect emerged gradually after the intervention, as fatigue recovered, and produced an optimal PAP at 12 min. A sharp deCIine was observed at 16 min, and the other two groups also showed a similar downward trend. The CON demonstrated a lower result than the pre-test at 16 min, indicating that the PAP induced by activation training gradually disappeared at this time. The non-dominant side exhibited a wave-like deCIine-rise-deCIine trend, which was attributed to the unequal musCIe strength between the non-dominant and dominant sides. The non-dominant side was found to be weaker and more prone to fatigue, while the dominant side was able to withstand greater loads and was less fatigued. Consequently, the fatigue effect of the organism exceeded the PAP at 4 min, resulting in a lower test result than the pre-test.

A comparable waviness was observed in the punching force, with the dominant side reaching its peak at 12 min in all three groups and exhibiting less fluctuation on the non-dominant side. However, no significant difference was found in the individual measurement time nodes. In terms of punching power, the 20VL on the dominant side demonstrated a notable increase in both the 8-min and 12-min time nodes. The 10VL on the non-dominant side exhibited a similar pattern, with a sharp increase and peak at 8 min. In addition to the differences in fatigue effect and fatigue recovery time under different VL, it is also essential to consider the changes in the physical function of the subjects under the fatigue state. When the speed and force of the punch remain unchanged, the coordination and stability of the body are negatively affected, which in turn impairs the kinetic chain’s conduction effect. This results in a reduction in punching power. The rationale behind the present study’s decision to increase the punching power test indexes is to address this issue.

The differing groups and time points for the optimal PAP between the dominant and non-dominant sides in this study were attributed to the uneven musCIe strength between the two sides of the subjects, which was a consequence of the characteristics of the boxing programme. This inconsistency in the stimulation of the training loads received by the musCIes on the dominant and non-dominant sides led to differences in the fatigue effect, as well as in the time point at which the fatigue effect appeared and disappeared. This renders it impossible to achieve the optimal PAP under the same training load bilaterally.

## 5 Limitation

The subjects in this study were all male. Further research is needed to determine whether the conCIusions drawn are also applicable to female boxers. This study only investigated the effects of PAP on the punching ability of boxers at different velocity loss thresholds from the perspective of athletic performance. It did not address the physiological mechanisms.

## 6 Conclusion

We found that activation training based on velocity loss induced a significantly better PAP than traditional activation training with fixed loads, which was more effective in improving boxers’ punching ability in a short period of time. The percentage of VL can be employed as a load modifying variable in strength training for the purpose of inducing PAP, which helps to improve the relevance and effectiveness of activation training through individualised, real-time monitored data feedback, applicable to the competition demands of high-level athletes.

Due to the interplay of load stimulus, fatigue effect and fatigue effect recovery time, we found that the PAP produced by different VL at different recovery time nodes differed and showed wave-like changes. We also found that there was a significant asymmetry between the punching ability of the dominant and non-dominant sides, with the punching ability of the dominant side being significantly better than that of the non-dominant side at different VL, and that the VL and time nodes at which the optimal PAP could be produced were not the same for both sides. The optimal effect was observed for the dominant side at the 12th minute of the 20% VL, while for the non-dominant side, the optimal effect was observed at the 8th minute of the 10% VL.

In practice, given that the dominant side has a greater punching ability and frequency of use than the non-dominant side in boxing events, it is recommended that a 20% VL be selected for activation and a 12-min interval in the pre-fight warm-up to achieve the best activation effect of the dominant side in order to maximise the short-term training benefit. In daily training, it is recommended to balance the dominant and non-dominant sides, but the results of the present study are only for the pre-competition warm-up, and its long-term training effect has not been confirmed, so further research is expected in the future.

## Data Availability

The raw data supporting the conclusions of this article will be made available by the authors, without undue reservation.
